# Supplementary material for: Are carbon emissions trading and green financial instruments synergistic? -Comprehensive quantitative research based on content analysis
Source: PLoS One. 2024 Mar 7;19(3):e0298601. doi: 10.1371/journal.pone.0298601 (PMC10919601; doi:10.1371/journal.pone.0298601)
Supplement: S1 Data — (ZIP) [file pone.0298601.s001.zip › Data/地级市Z_F工作报告.zip!/╣π╢1⁄2/╜¡├┼╩╨/└┤╫╘╣1⁄2╓┌║┼í╛┬φ┐╦╩2╛▌═°í┐_2012.docx]

各位代表：

　　我代表市人民政府向大会作政府工作报告，请予审议，并请政协各位委员和其他列席人员提出意见。

　　过去五年工作回顾

　　预计2011年全市生产总值达1780亿元，比上年增长13%:

　　地方财政一般预算收入达119.17亿元，比上年增长20.48%;

　　规模以上工业增加值达1130亿元，比上年增长19%;

　　全社会固定资产投资达760亿元，比上年增长21%;

　　社会消费品零售总额达760亿元，比上年增长16%;

　　外贸进出口总额达170亿美元，比上年增长18%;

　　2011年主要工作回顾

　　本届政府任期从2007年到2011年，五年来，市政府在省委、省政府和市委的正确领导下，在市人大及其常委会和市政协的监督支持下，深入贯彻落实科学发展观，加快转变经济发展方式，扎实推进经济社会建设和民生各项事业发展，全市经济综合实力跃上新台阶，2011年预计生产总值1780亿元，地方财政一般预算收入、规模以上工业增加值、固定资产投资、社会消费品零售总额、金融机构各项贷款余额等主要经济指标实现五年翻一番，较好完成了市第十三届人民代表大会、“十一五”规划和2011年确定的各项预期目标任务（见附件1）。特别是经过历届党委、政府和全市人民的共同努力，成功创建“全国文明城市”，标志着江门城市发展水平实现新跨越。

　　——过去五年，是实施《珠三角规划纲要》推进转型升级取得显著成绩的五年。

　　实施《珠三角规划纲要》工作在全省2010年度考评中获得总分第一，为实现“四年大发展”打下坚实基础。园区建设、大项目引进和战略性新兴产业培育取得重大突破。各级核心工业园区累计引进项目超千个，江门高新区晋升为国家级高新技术开发区。广东南车、台山核电、富华重工等大项目建设进展顺利，美的、海信、康师傅、普利司通、星辉造纸等大企业成功落户，成为省市共建绿色光源、轨道交通装备基地。着力提升自主创新能力，创建摩托车、半导体光电产品、机械装备等国家检测中心和检测重点实验室。以旅游、物流、金融为重点的现代服务业加快发展。“碉楼、温泉、海岛”等旅游品牌进一步打响，旅游总收入突破150亿元。新引进银行、保险、证券等金融机构38家，2家公司在境内挂牌上市，2家农信联社成功改制为农村商业银行。

　　——过去五年，是交通基础设施和宜居城乡建设取得重大突破的五年。

　　五年全市交通基础设施累计完成投资170亿元，高速公路通车里程达378公里，被列为国家公路运输枢纽城市。广珠城轨江门支线、江珠高速、江肇高速、滨江大道等建成通车，江顺大桥、江番及江珠北延线高速建设启动。促成珠中江在全省率先实现年票互认。崖门5000吨级出海航道整治工程顺利完成。城市基础设施建设力度加大，滨江新区建设全面铺开，启动区13项基础设施加快建设。天沙河引水增流工程投入运行。完成“三旧”改造2609亩。全市286公里省立绿道和228公里城市绿道全线贯通。生态环境进一步优化，全市已建成城镇污水处理设施30座，城镇生活污水处理率达75%以上，城市集中式饮用水源水质达标率100%。城市管理和绿化美化水平明显提升，高分通过“国家园林城市”复评。

　　——过去五年，是“三农”工作迈上新台阶的五年。

　　现代农业发展有新突破，五年新增87个农民专业经济合作组织，创建了全省首个国家现代农业示范区和粤台农业试验区，成功举办广东省第二届农运会、两届农博会、首届中国（江门）锦鲤博览会和新会陈皮文化节，台山市荣获“全国粮食生产先进单位”。农村基础设施建设步伐加快，“村村通自来水”工程有效推进，全市11.5万农村人口的饮用水不安全问题得到全面解决。五年完成农村公路硬底化1733公里，实现镇客运站、通车行政村候车亭全覆盖。城乡电力基础设施完成投资114.9亿元，农村电网实现“同网同价”。“农村信息直通车”覆盖所有行政村。

　　——过去五年，是文化社会各项事业全面进步的五年。

　　侨乡历史文化保护与传承、文化产业发展、文化设施建设成果丰硕。开平碉楼与村落成为广东省首个世界文化遗产，成功举办中央电视台“江门月·中华情”中秋晚会、华人嘉年华、世界江门青年大会等一系列打响江门城市品牌的重大活动。新建成江门五邑华侨华人博物馆、星光公园、城市规划展览馆、江门市青少年宫、江门广播电视中心等一批文化设施。成功创建广东省教育强市，中小学教师工资福利待遇“两相当”及代课教师问题得到全面落实和解决，100%解决外来人口子女接受义务教育问题，校安工程危房改造有效推进。医疗卫生服务机构实现城乡全覆盖，卫生机构床位数增长31%，新建农村卫厕17.86万户。我市体育健儿在北京奥运会和残奥会以及广州亚运会和亚残运会上勇夺三金三银一铜，全民健身活动蓬勃开展。获得“全国双拥模范城”五连冠和“全国人民防空先进城市”称号。扶贫开发“双到”工作取得阶段性成果。完成支援灾区抗震救灾和对口援建任务。侨务、外事、港澳、对台、民族、宗教、人口、计生、老龄、统计、审计、新闻、出版、档案、方志、气象、残联、红十字会等工作均取得新进步。

　　——过去五年，是市民群众得到更多实惠的五年。

　　把“创文”作为民生工程扎实推进，保障和改善民生的力度明显加大，五年来全市各级财政民生投入229.27亿元，年均增长22.13%。城乡居民收入不断提高，全市城镇居民人均可支配收入和农民人均纯收入五年分别增长72%和76%，分别达到23881元和9877元。就业再就业工作上新水平，在全省首创“千企扶千村”就业工程，五年全市新增就业岗位20.9万个，转移农村富余劳动力18.16万人。社会保障能力持续增强，城镇职工基本养老、基本医疗、工伤、生育保险参保人数基本实现翻一番，城乡居民基本医疗保险和新型农村社会养老保险实现全覆盖，启动完全被征土地农村居民基本养老保障试点工作。超额完成省下达的保障性住房建设任务。社会志愿服务队伍不断壮大。慈善事业蓬勃发展，投放善款近4亿元。公共交通不断完善，全市新增公交车691台。“平安江门”建设力度持续加大，共创建省“六好”平安和谐社区[1]218个。产品质量和食品药品安全监管整治取得实效。安全生产隐患治理成效显著，重特大事故得到有效遏制。

　　——过去五年，是服务型政府建设明显加强的五年。

　　推动机关作风改进和效率提高的一系列措施取得实效，“12345”政府服务热线覆盖全市，江门市新行政服务中心建成并投入使用。建立健全倒逼机制，政府执行力得到提升。自觉接受市人大及其常委会和市政协的监督，人大议案和建议、政协提案办理满意率分别达98.1%、99.8%。稳步推进“大部制”试点、简政强镇、审批制度改革，85%以上的审批权下放各市、区。落实信访维稳领导“一岗双责”和包案责任制，所有镇（街）建成综治信访维稳中心，有效解决一批涉及群众切身利益的问题。效能监察、廉政建设和反腐败工作进一步加强，查处一批违纪违法案件。

　　各位代表，过去五年我市经济社会发展取得了显著成绩，这离不开上级党委、政府和市委的正确领导，离不开市人大、市政协与各民主党派的大力支持，离不开全市人民、外来投资者和建设者以及驻江门军警部队的共同努力，离不开港澳台同胞、海外侨胞以及国际友人的关心帮助。在此，我代表市政府，向所有参与、支持和关心江门发展建设的各界人士，表示衷心的感谢！

　　回顾五年来的政府工作，我们积累了许多宝贵的经验，其中最为关键的是做到了“五个坚持”：一是坚持把建设现代产业体系、推进转型升级作为经济工作的主线；二是坚持把加快交通基础设施建设作为融入珠三角一体化的突破口；三是坚持把加强宜居城乡建设和“三农”工作作为统筹城乡发展的关键；四是坚持把以人为本、为民办好事实事作为政府工作的出发点和落脚点；五是坚持把转变作风、提高效能作为政府自身建设的主要抓手。这些好经验、好做法，我们将一以贯之坚持下去。

　　在充分肯定成绩的同时，我们也清醒地看到工作中还存在不少突出问题：制造业实力不够强，服务业在生产总值所占比重偏低，制约经济发展的体制问题仍然存在；以公共服务均等化统筹城乡发展的力度不够大；政府职能转变没有完全到位，办事效率与企业、市民的期望还有一定差距，等等。这些问题，我们将高度重视，采取有力措施加以解决。

　　今后五年发展目标和任务

　　今后五年，是我市深入实施《珠三角规划纲要》、加快转变经济发展方式的重要时期，也是我市经济社会蓄势待发的机遇期。今后五年政府工作的指导思想和主要目标是：深入落实科学发展观，全面贯彻市第十二次党代会精神，围绕“真抓实干，建设幸福侨乡”这一核心，坚持民生优先、生态优先，加快转变经济发展方式，全面推进经济、政治、文化、社会、生态文明建设，到2016年，努力实现全市生产总值、人均生产总值、地方财政一般预算收入、规模以上工业总产值比2011年翻一番，城镇居民人均可支配收入和农民人均纯收入分别达到40000元和16000元，努力把江门建设成为产业强市、宜居城市、文化名市、和谐家园。

　　未来五年，政府工作的主要任务是：

　　——推动经济大发展。

　　大力实施沿江边、沿铁路和高速公路边、沿海边的产业发展战略，加快形成“三边发展、三圈递进[2]”的产业布局框架。坚定不移地走新型工业化道路，加快先进制造业重点发展区建设，努力构建现代产业体系，切实做强制造业、做大服务业、做优大农业，实现产业集聚、结构优化、经济转型。以交通运输设备制造业、装备制造业、电子信息、精细化工、新材料五大产业为主体，构建先进制造业发展基本架构。坚持把招商引资作为经济发展的主攻方向，实施优质大项目带动、大平台支撑战略，加快核心园区建设，有效整合产业发展载体和工业用地，探索利用新型投融资方式完善园区配套设施，优化园区功能布局，以“三资融合[3]”新模式推动民营科技园发展，切实提升园区管理水平和承接产业能力。加大战略性新兴产业和央企、龙头民企、世界500强企业招商力度，提高招商引资的质量和水平。大力推进自主创新，实施创新和科研团队引进计划，加大创新人才培养和引进力度，重点培养和引进战略性新兴产业发展的中高级技术人才、领军人才和创新科研团队，努力创建国家级、省级技术创新平台。加快构建现代海洋产业体系，做大做强以海洋船舶等装备制造、电力能源、滨海旅游为主导的海洋产业，促进海洋经济又好又快发展。

　　——增创城乡环境新优势。

　　加快城乡基础设施建设、功能建设、环境建设，努力把我市建设成为布局更合理、功能更完善、交通更快捷、生活更便利、环境更优美、特色更鲜明的宜居宜业宜游生态型城市。优化城市功能布局，推进西江经济带和台山、开平、恩平统筹规划，实现功能互补。树立城市转型发展新理念，推进中心城市、中心城区、中心镇和一般小城镇协调发展，推动理想城市建设，提高城市化发展水平。坚持走资源节约型和环境友好型“两型社会”发展道路，建立健全法制环保长效机制，切实增强环保自觉意识，更加注重农村环境改善，大力发展低碳经济，加强生态建设，守住“绿水青山、蓝天白云”，实现人与自然和谐发展，进一步彰显江门“山水之中有城市、城市之中有山水”的独特魅力。

　　——提升侨乡文化软实力。

　　大力挖掘和弘扬侨乡文化，加快公共文化设施建设，建立公共文化服务体系，推进文化事业、文化产业发展，建设具有较强竞争力的文化产业体系，实现侨乡文化大发展大繁荣。深化包装、宣传、推介、营销侨乡文化，打响侨乡文化品牌，大力推动文化产业集约化、规模化、品牌化发展，打造千亿文化产业集群，重点发展印刷发行、传统工艺、文化旅游、动漫游戏、文娱演艺等行业。注重城乡历史风貌街区的保护和开发利用，打造一批文化新地标，塑造高品质的城市文化形象。积极搭建群众文体活动平台，深化文化惠民工程。提高教育现代化水平，提升城市文明程度和市民整体素质。

　　——构建和谐社会新格局。

　　坚持以人为本、民生为重，把保障和改善民生作为政府工作的出发点和落脚点，大力推进社会建设，提高社会管理水平，使改革发展成果惠及全市人民。立足江门作为农业大市的实际，把“三农”工作作为统筹城乡发展、推进公共服务均等化、促进社会和谐稳定的重点来抓。坚持富民优先，努力提高城乡居民收入水平。统筹城乡区域协调发展，推进教育、医疗卫生等基本公共服务均等化，建立健全城乡一体、全面覆盖、服务完善的社会保障和救助体系。加快构建诚信江门，营造公平公正、规范有序的社会环境。加大社会维稳和安全生产工作力度，维护江门良好的社会治安环境。

　　——树立实干政府新形象。

　　政府工作关键在真抓，关键在实干，关键在取得实效，必须进一步创新政府管理，优化政府服务，提升政府系统执行力、协同力、公信力。必须以改革创新的精神，加快转变政府职能，分类整合行政资源，坚持发挥好“12345”政府服务热线、民生热线等服务平台作用，着力提高行政服务中心运行效率和服务质量，进一步提升为企业、为群众、为基层服务的水平。必须坚持科学民主决策，切实保障市民群众的民主权利和合法权益。必须推进依法行政，提高政府运用法律手段管理经济和社会事务的能力。必须抓好廉政建设，完善源头防范、权力制约和内部监督等机制，坚决惩治和预防腐败。

　　2012年工作安排

　　今年是新一届政府承前启后、加快发展的开局之年，也是落实《珠三角规划纲要》、完成“四年大发展”目标任务的关键之年。政府工作的指导思想是：深入贯彻落实中央经济工作会议、省委十届十一次全会、市第十二次党代会精神，坚持“稳增长、扩内需、调结构、促改革、惠民生、保稳定”，加快转变经济发展方式，加大招商引资力度，加强社会建设和管理创新，大力保障和改善民生，推进幸福侨乡建设。

　　今年全市经济社会发展的主要预期目标是：生产总值增长13%，人均生产总值增长12%，固定资产投资增长18%，社会消费品零售总额增长16%，外贸进出口总额增长10%，地方财政一般预算收入增长13%；服务业增加值占生产总值比重达37%；研究与开发经费支出占生产总值比例1.2%；居民消费价格涨幅控制在4%左右；城镇居民人均可支配收入增长12%，农民人均纯收入增长12%；城镇登记失业率控制在3.2%以内；单位生产总值能耗下降和主要污染物减排完成省下达的目标任务。

　　对于今年的政府工作，我们既要紧抓江门新一轮大发展的机遇乘势而上，又要认清复杂形势的挑战沉着应对，切实增强紧迫感、责任感和使命感，凝心聚力，真抓实干，确保经济平稳健康可持续增长，稳中求进，稳中求好，把建设幸福侨乡各项工作落到实处。今年政府工作的重点是：

　　（一）加快转型升级步伐，确保经济平稳健康可持续增长

　　加快核心工业园区建设。着力“建大平台，招优质项目”，坚持土地节约集约利用，集中力量重点加快江门国家高新区、江沙示范园区、轨道交通装备产业园、银洲湖纸业基地、广海湾工业园区、江门产业转移工业园、鹤山工业城等核心园区建设。理顺江门国家高新区管理体制，探索“一区多园”发展模式，加快推进江沙示范园棠下片区和雅瑶片区统一连片规划开发建设。积极推进台山清洁能源（核电）装备产业园纳入省产业转移园区工作。

　　加速战略性新兴产业发展。抓好高端新型电子信息、高端装备制造、新光源、新能源、新材料等新兴产业发展和广东南车、台山核电、富华重工、美的、海信、康师傅、普利司通、星辉造纸等重大项目建设。积极争创全省海洋经济发展试点，以广海湾及川岛区域、银洲湖区域为重点，大力探索岛湾联动、岛海统筹的海洋开发模式，加快发展海洋经济。

　　加紧传统产业转型升级。运用高新技术、先进适用技术和信息技术提升改造机电、电子信息、纺织服装、造纸、食品、建材等传统产业转型升级，抓好李锦记、美达锦纶、泰宝涤纶、卡夫食品、西铁城LED、艾默生网络能源等企业增资扩产。大力推进“质量兴市”和“品牌兴市”战略，鼓励企业争创名牌和参与国际、国家、行业标准制（修）订。健全自主创新体系，加大公共技术服务平台建设力度，大力支持国家级、省级检测中心和企业技术中心建设。

　　加促现代服务业发展。重点发展旅游、物流、金融等现代服务业。高起点做好旅游规划，围绕打造世界文化遗产旅游区、温泉旅游度假区、现代滨海旅游度假区，大力整合全市旅游资源，推进滨海旅游产业园区规划建设，提升与港澳台、外省及国际旅游合作水平，做大做强旅游业。推进银洲湖、鹤山国际物流港、南新区等现代物流基地和绿色光源专业市场、江门义乌小商品城、江门时代广场、新会美吉特广场等商贸中心建设。支持金融改革创新，打造政银企合作平台，努力扶持企业上市，鼓励金融加强对产业发展的支持。

　　加大国内外市场开拓力度。稳定物价水平，增强居民即期消费能力。推进“农超对接”和平价商店建设，全市建立和完善区域商品配送中心2家，实现“农超对接”农民专业合作社15家，完成创建农资放心店20家。着力培育社区服务、教育培训、体育健身、养老保健、文化娱乐等新兴消费热点，进一步扩大休闲旅游、餐饮、商贸等传统消费，促进房地产市场健康发展。积极实施进出口市场多元化战略，扩大进口规模，大力开拓东盟、俄罗斯、南美、非洲等新兴出口市场。推进加工贸易转型升级，推动江门名优产品开拓国内外市场。

　　（二）加快基础设施建设，打造生态宜居城乡

　　推进大交通建设。抓好江顺大桥、广珠铁路、江番及江珠北延线高速、佛开高速扩建、江罗高速等重大交通基础设施项目建设，加快推进江门大道（广佛江快速通道江门段暂定名）、中开高速、325国道复线I工程（含鹤南西江大桥）、广佛江珠城轨、深茂铁路的前期工作，配合做好与港珠澳大桥的规划衔接工作。加大港口码头规划建设和岸线保护力度，加快推进广海湾港区的规划建设，争取崖门3万吨级航道整治等项目提前动工，抓好沿西江和崖门水道大型公用码头的建设研究工作。

　　推进城乡建设。进一步优化和完善城乡建设规划，提高控制性详细规划覆盖面。推进城市设计和“三规融合[4]”工作，塑造具有鲜明地域特色的城市风貌。坚持“科学开发、从容建设”，高标准、高规格推进滨江新区启动区重点基础设施建设，大力引进大财团建设大型城市综合体和中央商务区，启动滨江体育中心、小学、国家示范性中学和医院建设。加大“三旧”改造力度，同时保护好历史文化街区。抓好宜居试点创建工作，推进7个宜居城镇、14条宜居村庄试点建设。加快名镇名村建设，力争今年建成1—2个名镇、25个名村。以农村“五改[5]”和“三清[6]”工程为重点，抓好全市村庄整治工作，确保今年开展整治的村庄达629条、占全市行政村总数的60%。

　　推进环境保护和生态建设。大力实施“治污、护水、保山、建园”工程，为市民群众营造优美舒适的生活环境，迎接国家环境保护模范城市复检。严格落实节能减排目标责任制。大力发展循环经济，推进清洁生产。加快城镇污水处理设施建设和升级改造，年内完成江海生活污水处理厂二期管网建设，推进杜阮生活污水处理厂、棠下生活污水处理厂、高新区工业污水处理厂加快建设，全市城镇污水处理率达到80%以上。

　　加大城镇生活垃圾收集、清运力度，无害化处理率达85%。加强大气污染防治，探索推进将PM2.5纳入空气质量评价体系。实行最严格的水资源管理制度，加强对西江、潭江、天沙河等重点河段、河涌以及近岸海域环境的综合整治和保护。进一步推进天沙河截污工作，高标准启动天沙河沿岸绿化整治示范段建设。加大对自然山体和湿地绿地的保护力度，加快生态景观林带建设。依托城乡山水资源新建或改造丰乐山公园、星光公园二期、北新区体育公园、篁庄公园、怡福公园以及新会南湖公园、荔枝山公园、圭峰山绿护屏景区等一批社区公园、城市公园、郊野公园、森林公园和体育公园，完善已建公园的配套设施建设，为群众提供更多更好的休闲娱乐场所。继续完善绿道网功能配套，全市新建40公里城市绿道。力争今年建成区人均公园绿地面积达12.2平方米以上。

　　推进城市一体化建设。加快珠三角“五个一体化[7]”建设，进一步落实珠中江区域合作的30多项协议，推进《珠中江区域紧密合作规划》编制工作，积极推进珠中江城市（镇）供水同网工作，以扎实工作迎接省“四年大发展”验收。加快推进市区自来水、清洁卫生、生活垃圾、有线电视、公交等价格收费标准的同城一体化。统筹推动江会路片区开发。

　　（三）加强“三农”工作，推动农业大市向农业强市迈进

　　重点推进惠农支农政策落实。健全农业补贴制度和涉农资金监管制度，加强资金使用跟踪问效。完善财政支农与金融支农的配套政策。扶持发展农村集体经济，多渠道促进农民增收。强化农业行政执法，严厉打击坑农害农的不法行为。严格落实耕地保护制度。

　　重点推进农村基础设施建设。发挥政府投资引导作用，推动城乡交通、水利、电力、通讯、信息网络等基础设施对接，改善农村生产生活条件，尤其要加快推进民生水利建设，今年各级财政投入水利工程建设资金8.94亿元，比上年增长106%。加快实施“农田水利万宗工程[8]”和“双千”工程[9]建设，加快32宗万亩以上中型灌区改造工程和农村机电排灌工程、“五小”水利工程[10]建设，年内完成台山大隆洞河、鹤山沙坪河的治理。抓好“村村通自来水”工作，进一步提高全市行政村通自来水覆盖率、农村自来水普及率、农村生活饮用水水质合格率。

　　重点推进现代农业示范带动。加大江门市现代农业综合示范基地建设力度，推进开平国家现代农业示范区、粤台（江门）农业试验区和台山都斛万亩水稻高产示范田建设。不断提高农民组织化程度和农业产业化水平，今年新发展市级以上农业龙头企业3家以上，发展或改造市级农民专业合作示范社7家以上。加大“三品一标[11]”开发力度。办好第三届农博会。

　　（四）加强文化体系建设，塑造侨乡文化品牌

　　深化文化惠民工程。广泛开展群众性文体活动，大力推进文化下乡、进社区，实现农村电影放映、文化室、农家书屋、文化信息资源共享工程基层服务点覆盖每个行政村。加强文化基础设施规划建设，启动新图书馆、新文化馆、江门大剧院等标志性文化工程前期工作。

　　加强文化保护利用。进一步加大开平碉楼与村落等文化遗产和城乡历史风貌街区的保护利用力度，深入挖掘和整理非物质文化遗产项目。创新传承曲艺、武术、古典家具及陈皮、葵艺等民间特色文化。实施侨乡文艺精品工程，繁荣文艺创作，深层次彰显侨乡文化精神魅力和形象。

　　加快文化产业发展。鼓励和支持动漫、广告等文化产品的创意设计和制作，大力发展现代传媒、印刷发行、文娱演出等产业，促进文化与科技、创意、商贸、旅游、制造业等融合发展。办好第二届江门侨乡动漫节。

　　（五）加大保障和改善民生力度，推进社会建设和管理创新

　　今年各级财政预算民生投入76.3亿元，比上年增长24.29%。全市各级要大力推进以保障和改善民生为重点的社会建设，建立完善“创文”工作长效机制，为广大老百姓办好10件民生实事（见附件2）。

　　提升社会保障水平。建立起“普惠型”的城乡基本养老保险制度体系，做好完全被征土地农村居民养老保险与新型农村社会养老保险制度、城镇居民社会养老保险与城镇职工基本养老保险制度的衔接。全市新增保障性住房5000套。提高社会救助水平，全市城乡低保标准提高到2500元/年以上，确保五保供养标准达到农民人均纯收入的60%以上。

　　促进就业与培训工作。今年全市城镇新增就业岗位4.5万个。抓好农民工入户和技能培训工作，继续深化“千企扶千村”就业工程，全市培训农村劳动力4万人，转移农村劳动力就业2万人。深入推进校企合作，扩大“双零[12]”合作规模，实现技工学校和企业“双赢”。继续做好“三支一扶[13]”、大学生村官服务工作，鼓励高校毕业生面向基层就业。

　　优先发展教育事业。启动创建省县域教育现代化先进市（区）工作。加强教育资源整合，进一步提高“普九”、“普高”的质量和水平。加快普及学前教育，重点发展农村学前教育和推进乡镇中心幼儿园建设，全市学前三年入园率达93%，全面实施学前残疾儿童三年免费教育。落实好农村义务教育学生营养改善计划的试点工作。推进高等教育、高职教育向产学研纵深发展和更好地为地方服务。抓好中等职业技术教育战略性布局结构调整工作。推动和规范民办学校办学行为。

　　优化医疗卫生服务。抓好国家卫生城市复检工作。进一步深化医药卫生体制改革，加强全市三级医疗卫生服务体系建设，着力提升基层卫生服务能力和水平，促进城乡卫生服务均等化。积极探索公立医院改革试点工作。进一步完善全市医疗急救体系。积极防控重大传染病。

　　保障群众便利出行。进一步推行公交优先，加大公共交通投入，今年市区更换和新增公交车220标台。抓好市区主干道、高速公路出入口的建设改造以及重要地段、重点路口的绿化改造升级，进一步完善轻轨站场配套设施建设。年内完成东华大桥、胜利大桥、胜利路人行天桥和胜利南路等城市路桥建设，进一步畅通城市交通。全市完成农村公路建设150公里。

　　探索社会管理创新。完善社区建设，提高社会管理效能，全市完成60个农村社区服务中心建设，实现15%行政村建立农村社区服务中心。壮大社工服务队伍，进一步发展义工事业。加强城市管理和市容市貌整治，新改造市区农贸市场10个。探索和完善户籍管理制度改革工作，逐步建立城乡统一的户口登记制度，加强流动人口管理与服务工作。抓好信访维稳工作，落实领导“一岗双责”和包案责任制，发挥镇（街）综治信访维稳中心作用，妥善处理好人民群众的合理诉求，提高社会矛盾纠纷排查化解工作能力。

　　强化平安江门建设。严格落实党政领导安全生产“一岗双责”，建立安全生产长效机制，坚决遏制较大以上事故发生。建立健全覆盖城乡的公共突发事件应急处置体系，提高政府有效应对自然灾害、事故灾难以及公共卫生、食品药品安全、社会安全、环境安全事件能力和群众自救互救能力。加强社会治安监控系统建设，全市新安装治安视频监控摄像头2057个、治安视频卡口106个，推动社会力量参与治安监控系统建设，提升社会治安监控能力。巩固平安和谐社区和民主法治村（居）创建成果。

　　此外，要确保完成省下达的“双到”扶贫工作、人口计生任务。维护妇女儿童合法权益。做好新生代华侨的侨务工作。加强国防教育，完善国防动员体系，落实“双拥”和优抚安置工作。继续做好民族、宗教、外事、港澳、对台、老龄、人防、统计、审计、档案、方志、气象、残联、红十字会等工作。

　　（六）加大政府自身建设力度，提高服务效能

　　做好今年各项工作，是对政府施政能力的考验。我们要切实转变观念、改进作风、真抓实干，推进政府服务更便利、更优质、更高效、更贴心。

　　着力提高为企业、为群众、为基层服务水平。政府必须爱护企业、服务企业，大力打造民营企业和中小企业融资、综合审批、用工及人力资源、商会行业协会“四大服务平台”，实施民营企业腾飞计划。建立完善企业联合会与涉企职能部门的沟通机制，搭建涉企信息和惠企政策宣传发布平台。开通江门市政企通信息服务平台，完善重点企业直通车服务制度。设立中小企业融资平台，完善企业征信系统建设，积极扶持企业上市，为企业、商会行业协会提供“一站式”融资服务。加强用工培训服务，鼓励企业招用本地劳动力。畅通民意表达渠道，完善“12345”政府服务热线办理机制，扩大公众对政府工作的有序参与。加强电子政务建设，更大范围推行并联审批和网上审批，提高办事效率。推进多层次、全方位的服务网络建设，全市所有街道都建有“一站式”政务服务中心。深入推进政务公开。加大对各项便民、利民措施的宣传和指引，方便群众办事，主动接受群众监督。

　　着力提高政府执行力、协同力、公信力。坚持依法行政，加快法治政府建设。加强政府管理创新，坚持推进重大项目、重大活动、民生实事以及市委、市政府重大决策和重大工作等倒逼机制，以时限倒逼进度，以目标倒逼责任，把工作压力传送到每个环节和每个岗位，狠抓各项工作的落实。主动接受人大监督，积极支持人民政协履行政治协商、民主监督、参政议政职能，提高人大代表议案、建议和政协委员提案办理水平。强化绩效考核，加强效能监察、行政问责。

　　着力推进重点领域和关键环节改革。积极发挥政府在改革中的主导作用，突出推进培育发展和规范管理社会组织、住房保障制度和医疗保障制度等民生领域改革、市场经济体制改革等重点改革。以大部制改革为契机，大力推进市直有关部门的专项改革工作，理顺行政管理体制。进一步稳妥推进财政管理体制改革、行政审批制度改革、简政强镇改革。扩大政府购买服务的试点，建立社会组织购买政府服务的准入和退出机制，加强对政府购买服务的监察评估。

　　着力提高廉政建设水平。全面落实廉政建设责任制，规范各级领导干部从政行为，加强惩治和预防腐败体系建设。进一步强化“三公”经费[14]管理。加强对行政权力运行的全过程、全方位监督。规范工程项目招投标、国有土地使用权出让、公共资源交易、政府采购、公务消费等行为。继续加大案件查处力度，严惩腐败分子，以反腐倡廉的实际成效取信于民。

　　各位代表，未来五年政府工作艰巨而光荣、任重而道远，让我们深入贯彻落实市第十二次党代会精神，团结带领全市人民，继续解放思想，开拓创新，真抓实干，为建设幸福侨乡而努力奋斗！
